# Case Report: Niraparib as Maintenance Therapy in A Patient With Ovarian Carcinosarcoma

**DOI:** 10.3389/fonc.2021.603591

**Published:** 2021-12-06

**Authors:** Jie Qing Zhang, Bing Bing Zhao, Mao Mao Wang, Li Li

**Affiliations:** Department of Gynecologic Oncology, Guangxi Medical University Cancer Hospital, Nanning, China

**Keywords:** niraparib, ovarian carcinosarcoma, maintenance therapy, case report, gynecologic oncology

## Abstract

Ovarian carcinosarcoma (OCS) is a rare, highly aggressive and rapidly progressing malignant tumor with an extremely poor prognosis. So far, due to the low incidence of OCS, there are no large-scale prospective studies exploring the standard care of OCS patients. There is no uniform and effective treatment for OCS. Within the development of precision medicine, targeted therapies (such as PARP inhibitors) have been widely used in epithelial ovarian cancer and various other solid tumors. Here, we report a BRCAwt patient with advanced OCS who experienced a second and a third cytoreductive surgery in June 2017 and October 2019 and has been on niraparib maintenance therapy for more than 20 months after receiving second-line and third-line chemotherapy.

## Background

Ovarian carcinosarcoma (OCS), also known as ovarian malignant Mullerian mixed tumor (MMMT), is a malignant mixed mesodermal tumor with malignant epithelial and sarcoma components in the tissue; it is not common in clinical practice but highly malignant, accounting for 1% to 2% of all ovarian malignancies (1). OCS is more common in postmenopausal women aged 60-70 years without typical clinical manifestations, and most patients are diagnosed in an advanced stage with a poor prognosis and an average survival time of less than 1 year (2). At present, there is no uniform and standardized diagnosis and treatment plan for OCS. According to the NCCN guidelines, clinicians can follow the principles of epithelial ovarian cancer in OCS cases, and a comprehensive treatment of such malignant tumors is also recommended (3).

Poly (adenosine diphosphate-ribose) polymerase (PARP) inhibitors, such as niraparib, olaparib and rucaparib, had been approved as a maintenance therapy in epithelial ovarian cancer patients in remission following platinum-based chemotherapy (3). A recent case report showed a significant decrease in tumor burden in a 67-year-old patient with stage IIC OCS when treated with the PARP inhibitor olaparib (4). Clinical trials of PARP inhibitors have been extended to various solid tumors such as prostate cancer, gastric cancer, and head and neck cancer, with some obtaining good preliminary data and treatment response.

Here, we report a case of niraparib use in a 55-year-old woman diagnosed with OCS without BRCA gene mutation. She experienced OCS recurrence just half a year after 8 cycles of chemotherapy in 2017. Pathological findings showed components of serous adenocarcinoma and carcinosarcoma in the recurrence samples. The patient was commenced on niraparib following the second-line chemotherapy and remained in remission with good therapeutic response. To our knowledge, there are no published cases of successful use of niraparib as maintenance therapy in a patient with OCS.

## Case Presentation

The Fifty-three years old postmenopausal women presented at the Department of Gynecology in Guangxi Medical University Cancer Hospital with a ten-days history of abdominal pain and abdominal distension on May 25, 2016. She suffered breast cancer and had a Breast-conserving surgery followed by radiotherapy and chemotherapy in 1997, and has been receiving hormone therapy (tamoxifen po) irregularly since then. She was born in Qinzhou on April 10, 1963 and almost always lived in Nanning. Her living and working conditions were good. No bad personal habits or customs. Menstrual onset was 14 years old. The period lasted from 3 to 5 days, and its cycle is about 27 to 28 days. The age of menopause was 50 years old. She had pregnancy twice, once nature production, induced abortion once. She had no family history of malignancy, hypertension or diabetes mellitus. Physical examination showed no abnormity in lung and heart. Superficial lymph nodes were not enlarged. Shifting dullness were positive. Gynecological examination revealed a large palpable, fixed, non-tender masses in the pelvic region, which is about 15*10*10cm with slight tenderness, pressure recrectum. Cancer antigen 125 was elevated, at 171.2 U/mL. CT showed a mixed cystic solid tumor (largest level, 9.8 cm×8.9 cm×11.9 cm) in the pelvic cavity with large amounts of abdominal and pelvic effusion and multiple peritoneal metastases, the largest being about 2.0 cm×1.4 cm.

The patient underwent primary debulking surgery in May 18 2016. The right ovary increased by about 12 cm× 10 cm× 9 cm, with brittle, tumor rupture and bleeding, necrosis, adhesion to the lateral wall, and rectal fossa peritoneum. The left ovarian surface seemed normal. The anterior wall of the uterus and the reflexed peritoneum of the bladder were filled with small nodules of varying sizes. The diaphragm surface was smooth, and no visible lesions were found in the liver, gallbladder and kidneys. Para-aortic and pelvic lymph nodes were slightly enlarged. The deep red bloody ascites was about 5000ml.

**Figure 1:**
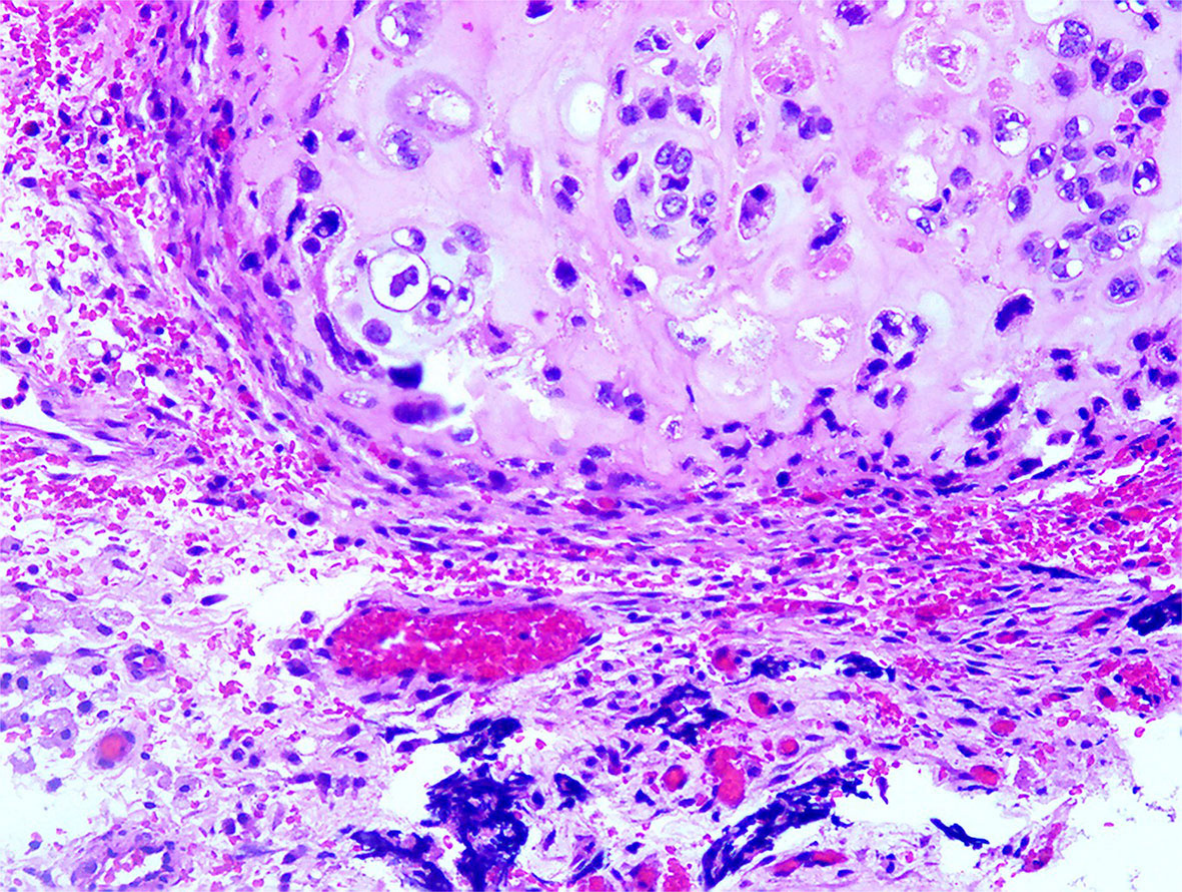
Sarcoma component (×100).

Pathology report revealed a malignant Mullerian mixed tumor (carcinosarcoma) (**Figure 1**).

Immunohistochemistry showed that the tumor tissue was CKpan+、CK7+、EMA+、PAX-8 + and CK20-. The sarcoma tissue was Vimentin+ and WT-1 partially positive. The carcinosarcoma was ki-67+ by about 98% (+).

Surrounding tissues (both the fallopian tubes and the anterior wall of the rectum) were extensively invaded by the tumor. The left ovary was normal. Serous papillary carcinoma was planted (BC16.536) in the appendix muscle layer and omentum. A total of 19 lymph nodes with reactive hyperplasia were dissected, including 7, 5, 2 and 5 in the left pelvis, right pelvis, pelvis and abdominal aorta’s vicinity, respectively. The patient was diagnosed with right ovarian malignant mullerian mixed tumor (carcinosarcoma) IIIC and administered the docetaxel plus carboplatin regimen for 3 cycles. Because of intolerance, the patient was switched to the epirubicin plus cisplatin regimen for another 5 cycles until December 22, 2016.

However, recurrence was detected half a year later. MRI scan showed multiple tumors in the pelvic cavity, vaginal stump, and bilateral iliac vessels, as well as around the rectum on June 5^th^, 2017. The patient underwent 2 cycles of ifosfamide plus oxaliplatin on June 22 and July 27, 2017. CT on Aug 23, 2017 showed unevenly thickened and partially nodular peritoneum. The largest size was about 2.1 cm×1.1 cm. The kidneys and ureters had slight hydronephrosis. On August 30, 2017, bilateral ureteroscopy and ureteral stenting were performed under intravenous anesthesia, and secondary cytoreductive surgery was performed on August 31 2017. The tumor sample was submitted to the BRCA test after surgery.

On Sep 16, 2017, the patient received ifosfamide plus oxaliplatin for first cycle chemotherapy after surgery, Grade IV myelosuppression occurred; the general situation was improved after platelet transfusion and systematic treatment. She then further refused chemotherapy. In September 2017, the BRCA test was negative.

The Patient was not willing to receive chemotherapy. Currently, it is almost impossible to cure recurrent ovarian cancer. In March 2017, the US FDA approved niraparib maintenance therapy for platinum-sensitive ovarian cancer, including the BRCA wild-type population. There are epithelial and sarcoma components in OCS, but no relevant clinical trials have been performed in China. We thought it was worth applying niraparib in such an advanced OCS case.

**Figure 2:**
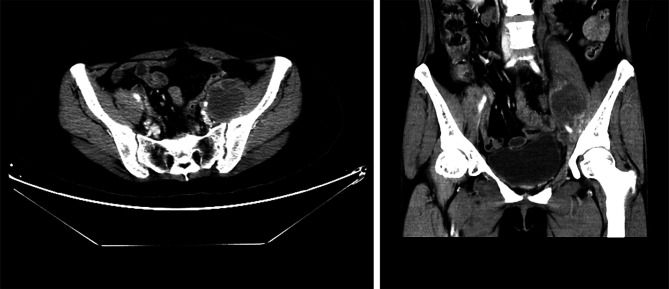
Pelvic MRI on April 1, 2019. Coronal view showing an anterior abdominal wall metastasis after niraparib administration for 20 months.

Niraparib is not approved in Mainland China and luckily, the patient had the drug from Europe and started taking it at 300 mg daily since October 2017. She developed severe thrombocytopenia after two months of niraparib treatment, with a platelet count of 43×10^9^/L. As a result, niraparib was discontinued for 4 weeks to allow hematological recovery. It was restarted at a reduced dose of 200 mg daily. On May 9, 2018, she had another episode of Grade 3 thrombocytopenia. After a two-week drug discontinuation, blood test was back to normal, and the patient has been on niraparib at 100 mg daily since then. On April 1, 2019, 20 months after the secondary cytoreductive surgery, MRI showed an anterior abdominal wall metastasis, as a locally isolated lesion of 2.4 cm×0.9 cm (**Figure 2**). The patient declined surgery until CT discovered 2 abdominal wall metastases (5.4 cm×4.6 cm and 2.5 cm×2.0 cm respectively) on October 28, 2019. Abdominal wall mass resection was performed on October 28, 2019, and 5 cycles of oxaliplatin at 200 mg combined with albumin paclitaxel at 400 mg were administered in November 2019-April 2020. Bevacizumab was added to the last cycle of chemotherapy.

## Outcome and Follow Up

**Figure 3:**
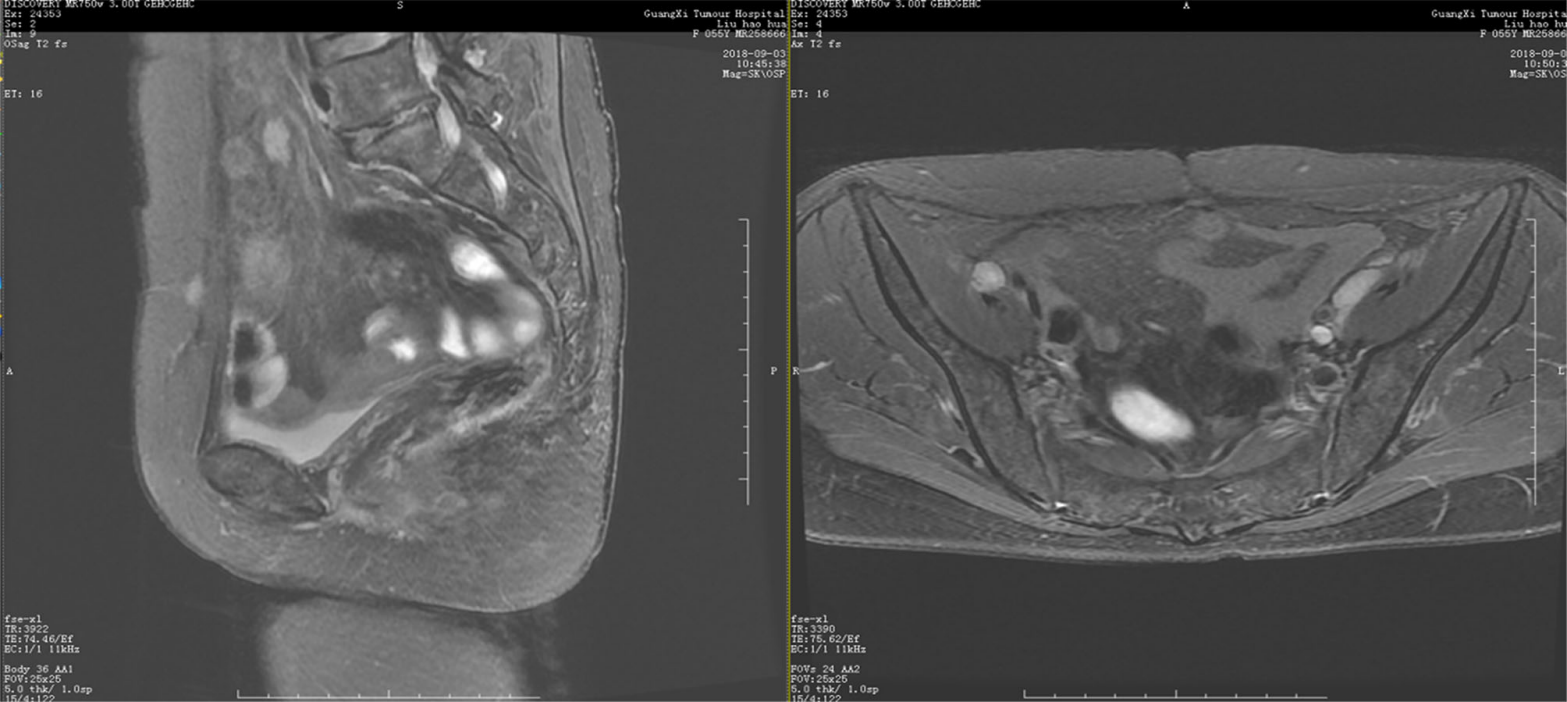
Pelvic MRI on September 3, 2018. Coronal view showing no new lesions after niraparib administration for one year.

**Figure 4:**
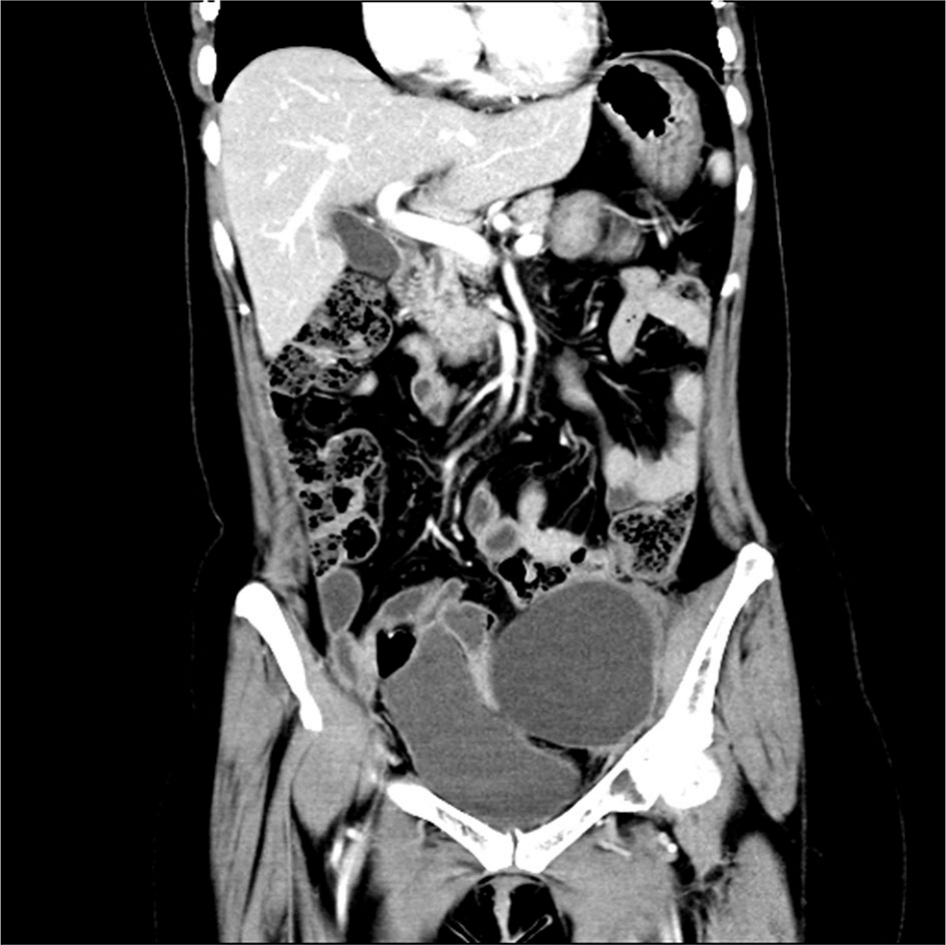
Pelvic MRI on June 5, 2020. No recurrence was found.

This 55-year-old woman otherwise tolerated niraparib very well. No significant tumor progression was observed. She is currently active, with normal quality of life. On December 7, 2017, CT showed no new lesions in the pelvic cavity after the second operation. Her condition was stable. Ileostomy and ileal partial resection with intestinal adhesion lysis were performed On December 14, 2017, and the patient recovered very well after surgery. MRI showed no disease progression at 13 months after the second surgery (**Figure 3**). After the most recent surgery, the patient’s general condition was good, CA125 levels were maintained at low levels(3.4 U/mL at June 4, 2020). The patient has been on niraparib plus bevacizumab maintenance therapy since April 2020, and the latest CT showed no sign of recurrence (**Figure 4**).

## Discussion

Ovarian carcinosarcoma is not common in clinical practice; however, it recurrence rate is about 70% and the tumor usually recurs fast, with a poor prognosis. Patients usually has multiple metastases in the abdominal and pelvic cavities, liver and lung, with a five-year-survival rate of 21% (5). So far, there is no large, randomized prospective study exploring a standard care in OCS patients due to low tumor incidence. Multiple retrospective studies suggested that a satisfactory cytoreductive surgery and chemotherapy are the most effective treatments. OCS is highly invasive with a wide range of lesions, involving multiple organs in the pelvis, and surgical treatment is quite challenging. At present, clinicians generally follow the principles of ovarian epithelial cancer chemotherapy in OCS patient; however, the sensitivity of OCS to chemotherapy differs from that of ovarian epithelial cancer. The optimal chemotherapy regimen for OCS remains undefined.

Poly (ADP ribose) polymerase (PARP) inhibitor-based cancer therapy selectively targets cells with deficient homologous recombination repair. It has been demonstrated that PARP inhibitors are effective in patients with deficient homologous recombination, especially in BRCA1/2 mutant individuals (6–13). PARP inhibitors significantly prolong progression free survival (PFS) in patients with advanced ovarian cancer. Niraparib, olaparib and rucaparib have been approved for maintenance therapy in platinum sensitive recurrent ovarian cancer.

In 2015, more data from study19 (9) showed that olaparib does not appear to benefit ovarian cancer patients with wild-type BRCA and homologous recombination repair, but may benefit non-BRCA-related HRD patients. The SOLO-2 study (10), a phase III trial including a larger population with BRCA mutations performed several years later, confirmed the benefit of olaparib maintenance treatment in platinum-sensitive, relapsed, high-grade serous ovarian cancer. However, whether patients with wild-type BRCA and homologous recombination repair could benefit from olaparib maintenance therapy remains unknown.

In 2016, ARIEL2 (11) included 204 patients with mutant and wild-type BRCA and loss of heterozygosity (LOH)-high platinum-sensitive ovarian carcinomas treated with rucaparib, and progression-free survival was longer than in cases with wild-type BRCA LOH-low carcinomas. However, ARIEL2 was a single arm study, and the benefits in wild-type BRCA cases and patients with low LOH have not been verified. The ARIEL3 (12) phase III trial enrolled 564 patients with platinum-sensitive recurrent high-grade serous or endometrioid ovarian carcinoma. Rucaparib significantly prolonged PFS in patients with a known genomic or somatic BRCA mutation (16.6 months for rucaparib *vs* 5.4 months for placebo; HR=0.23; 95%CI 0.16 to 0.34). In patients with HRD, defined as mutant or wild-type BRCA with high LOH, the values were 13.6 and 5.4 months for rucaparib and placebo, respectively (HR=0.32; 95%CI 0.24 to 0.42). However, there was no independent analysis in the wild-type BRCA/low LOH (HRD-) group. Therefore, the benefit of rucaparib in the HRD- group remains unknown.

The NOVA study (13), a phase III, randomized, double-blind clinical trial, included 553 patients with relapsed ovarian, fallopian, or primary peritoneal cancer, with 203 in the germline BRCA mutation (gBRCA) cohort and 350 in the non-gBRCA cohort; the niraparib group had significantly longer median PFS duration compared with the placebo group, with 21.0 *vs*. 5.5 months in the gBRCA cohort (HR=0.27; 95%CI 0.17-0.41), *versus* 9.3 *vs*. 3.9 months in the overall non-gBRCA cohort (HR=0.45; 95%CI 0.34-0.61 (P<0.001 for all three comparisons). Surprisingly, patients with HRD-negative tumors also benefited from niraparib treatment (median PFS, 6.9 *vs*. 3.8 months).

To the best of our knowledge, there is no current case report of PARP inhibitor administration for the treatment of patients with OCS. The current patient experienced platinum resistance-associated recurrence in early 2017. She was wild-type BRCA and intolerant to chemotherapy; treatment options are quite limited, and there was no available HRD test in China in 2017-2019. There is no high-level evidence demonstrating the benefit of olaparib and rucaparib maintenance in the wild-type BRCA and HRD- population. The NOVA trial demonstrated the benefit of niraparib in all-comer individuals among patients with recurrent ovarian cancer. We thought it was worth applying niraparib in such a challenging OCS case. After 2 years of follow up, the patient was generally in good health, with acceptable quality of life and no disease progression.

QUADRA (14), a phase II single-arm study, is underway to evaluate the safety and efficacy of niraparib in patients with advanced recurrent HGSOC who have received three or four previous chemotherapy regimens. This study met the primary endpoint, with an ORR of 28% in HRD-positive population. The median PFS in this population was 5.5 months. The median OS was 26 months in the BRCAm population, 19.0 months in the HRD-positive population. the Quadra study observed clinical benefits in each clinical subgroup and biomarker subgroup.

In 2020, some laboratories in China could conduct HRD tests. BGI genomics carried out 4 HRD tests, assessing archive tissue sections from surgery in 2016, 2017& 2019. In this patient, 3 tests failed (low DNA quality and quantity of the FFPE samples) and one positive result was detected in the surgical specimen of 2019, LOH, TAI and LST score is 25, 20 and 38 respectively, the ultimate HRD Score is 56.57 after ploidy correction. Homologous Recombination Deficiency may be one of the reasons why niraparib was effective in the current ovarian carcinosarcoma patient.

Although the patient experienced another relapse in 2019, considering that she had a platinum-free interval of up to 20 months, we re-administered platinum-based chemotherapy and bevacizumab plus niraparib maintenance therapy. We continue to follow up the patient, evaluating the extent of benefit from our medical plan.

## Conclusions

Ovarian carcinosarcoma is highly malignant, and most cases are diagnosed at an advanced stage. There is no consensus on the diagnosis and treatment of OCS, and survival time is very short with limited treatment options. In general, given the strong evidence that ovarian carcinosarcoma has a worse response to the chemotherapy treatment for epithelial ovarian cancer, alternate regimens need to be explored to improve post-operative PFS and OS.

The patient experienced a rapid recurrence after the first platinum based chemotherapy (defined a platinum resistant recurrent) and not tolerated to 2nd line chemotherapy. We believe if we doing nothing and just watching that the patient were going to experience a rapid disease progression and death in a very short period. We choose to use niraparib as a salvage treatment. The final result for the single case is good, niraparib administration maintained the current patient in remission for over 20 months, in the meanwhile she has received treatment of niraparib monotherapy only, and she currently enjoys a good quality of life and shows a very good functional status and proved that we might have a chance to do a larger trial in OCS patients for niraparib treatment or maintenance setting. This is an example of successful individualized treatment based on limited high-level medical evidence. Large prospective randomized controlled studies are needed to explore whether niraparib could constitute a new option for maintenance treatment in ovarian carcinosarcoma.

## Data Availability Statement

The original contributions presented in the study are included in the article/Supplementary Material. Further inquiries can be directed to the corresponding author.

## Ethics Statement

The studies involving human participants were reviewed and approved by the Ethics Committees of the Guangxi Medical University Cancer Hospital. The patients/participants provided their written informed consent to participate in this study.

## Author Contributions

LL and JZ carried out the studies, participated in collecting data, and drafted the manuscript. BZ and MW performed the statistical analysis and participated in its design. All authors contributed to the article and approved the submitted version.

## Conflict of Interest

The authors declare that the research was conducted in the absence of any commercial or financial relationships that could be construed as a potential conflict of interest.

## Publisher’s Note

All claims expressed in this article are solely those of the authors and do not necessarily represent those of their affiliated organizations, or those of the publisher, the editors and the reviewers. Any product that may be evaluated in this article, or claim that may be made by its manufacturer, is not guaranteed or endorsed by the publisher.
